# Examining the association between loneliness and emergency department visits using Canadian Longitudinal Study of Aging (CLSA) data: a retrospective cross-sectional study

**DOI:** 10.1186/s12877-022-02763-8

**Published:** 2022-01-22

**Authors:** Stephanie A. Chamberlain, Rachel Savage, Susan E. Bronskill, Lauren E. Griffith, Paula Rochon, Jesse Batara, Andrea Gruneir

**Affiliations:** 1 Department of Family Medicine, Faculty of Medicine & Dentistry, University of Alberta, 6-50 University Terrace, Edmonton, Alberta T6G 2T4 Canada; 2 Women’s College Research Institute, Women’s College Hospital, Toronto, Ontario Canada; 3 ICES, Toronto, Ontario Canada; 4 Institute of Health Policy, Management & Evaluation, Dalla Lana School of Public Health, University of Toronto, Toronto, Ontario Canada; 5 Sunnybrook Research Institute, Sunnybrook Health Sciences Centre, Toronto, Ontario Canada; 6 Department of Health Research Methods, Evidence, and Impact, Faculty of Health Sciences, McMaster University, Hamilton, Ontario Canada; 7 Division of Geriatric Medicine, Department of Medicine, University of Toronto, Toronto, Ontario Canada

**Keywords:** CLSA, Loneliness, Emergency department visits

## Abstract

**Background:**

Loneliness is a public health concern and its influence on morbidity and mortality are well documented. The association between loneliness and emergency department visits is less clear. Further, while sex and gender-related factors are known to be associated with loneliness and health services use, little research looks at the relationship by gender. Our study aimed to estimate the association between loneliness and emergency department use in the previous 12 months. We aimed to determine if this association differed based on gender identity and gender-related characteristics.

**Methods:**

We used a retrospective cohort study design to analyze population-based survey data from the Canadian Longitudinal Study on Aging (CLSA). We analysed data from the baseline and follow-up 1 survey respondents (2015-2018) from both the tracking (telephone interviews) and comprehensive (in-home data collection) cohorts (*n*=44816). Loneliness was assessed using a dichotomous measure (lonely/not lonely) from a validated scale. Emergency department visits were dichotomous (yes/no) by self-reported emergency department use in the 12 months prior to the survey date. Multivariable logistic regression analyses using analytic weights examined the association between loneliness and emergency department visit, controlling for other demographic, social, and health related factors.

**Results:**

We identified 44,413 respondents to the baseline and follow-up 1 survey. The prevalence of loneliness in our sample was 23.1% (n=10263). Of those who had been to the emergency department in the previous year, 27.2% (n=2793) were lonely. Lonely respondents had higher odds of an emergency department visit (aOR: 1.13, 95% CI: 1.05-1.21), adjusted for various demographic and health factors. Loneliness was associated with emergency department visits more so in women (aOR: 1.15, 95% CI: 1.05-1.25) than in men (aOR: 1.10, 95% CI: 0.99-1.22).

**Conclusions:**

In our study, loneliness was associated with emergency department visits in the previous 12 months. When our analysis was disaggregated by gender, we found differences in the odds of emergency department visit for men, women, and gender-diverse respondents. The odds of ED visit were higher in women than men. These findings highlight the general importance of identifying loneliness in both primary care and hospital. Care providers in ED need resources to refer patients who present in this setting with health issues complicated by social conditions such as loneliness.

**Supplementary Information:**

The online version contains supplementary material available at 10.1186/s12877-022-02763-8.

## Background

Loneliness is increasingly recognized as a major health issue, offering a unique opportunity to build concerns for social connection, community, and caring into public health [1]. As individuals get older, they are at heightened risk of loneliness because of age-related transitions including shrinking social networks, widowhood, and retirement [2, 3]. Loneliness itself is influenced by age, sex (biological) and gender-related (socio-cultural) characteristics and these factors are also associated with health service use [4]. Population based studies can be a useful way to look at issues of loneliness, risk factors and use of health services; although sex is often included as a covariate, few analyses are disaggregated by sex and even fewer consider the influence of gender identity or gender-related characteristics [4]. Factors that predict loneliness and reporting of loneliness often differ between men and women [5]. Social problems such as loneliness may have an effect on older adults’ use of health services and their health outcomes. Individual studies have shown some evidence that loneliness is associated with hospital visits [6, 7], and loneliness has been found to be a predictor of early and frequent returns to the emergency department (ED) [8]. Whereas other research has found loneliness to be associated with a greater number of physician visits, but not ED visits or hospitalization [9, 10]. The mechanisms through which loneliness impacts health service use are not entirely clear, with some studies finding both under-use and over-use of acute care and primary care services [7, 11–13]. As the number of older adults increases, research identifying how loneliness may contribute to service use in different populations is urgently needed.

Emergency departments (ED) are a critical site of care [14, 15]. Older adults are one of the largest patient populations seen in this setting and compared to younger patients they remain in EDs for longer lengths of time and are at heightened risk of adverse health outcomes [16–18]. Adverse health outcomes can include pressure ulcers, delirium, and increased likelihood of developing new or worsening disability. EDs are the most common entryway into the hospital system and hospitals place older adults at unique risk given their intersecting and complex care needs that can include physical and cognitive impairments and social problems [15, 19]. Identifying the relationship between loneliness and ED use is essential because it contributes to optimizing service use in older adults by ensuring individuals receive the right care in the right setting. This may improve the quality of care and quality of life of older adults. Our overall study objective was to estimate the association between loneliness and ED use in a 12-month period using follow-up 1 survey data from the Canadian Longitudinal Study on Aging (CLSA). Our secondary objective was to determine if this association differed based on gender identity and gender-related characteristics.

## Methods

We used a retrospective cross-sectional study design to analyse population-based survey data from the Canadian Longitudinal Study on Aging (CLSA) [20]. At the start of this study, the CLSA had collected two waves of data referred to as baseline (2011-2014, including a maintaining contact survey, 2013-2015) and the first follow-up (2015-2018). The CLSA consists of two cohorts: Tracking and Comprehensive. The Tracking Cohort (baseline, n=21,241) consists of an age- and sex-stratified random sample of community-dwelling Canadians aged 45 years and older who completed a computer-assisted telephone interview. The Comprehensive Cohort (baseline, n=30,097) is a stratified (age, sex) random sample of individuals 45 years and older who live within 25 to 50 km of a CLSA data collection site and took part in in-home interviews and provided biological data at CLSA data collection sites [20, 21]. A common set of core questionnaire data are collected from both cohorts. The CLSA uses the following exclusion criteria: individuals unable to respond in either English or French, persons who are cognitively impaired at the time of recruitment, those living in the three territories, full-time members of the Canadian Armed Forces, individuals living in long-term care homes at baseline, and persons living on reserves and settlements. Informed consent was obtained from all CLSA participants prior to data collection.

### Study sample

Our sample included participants in the first follow-up survey of the CLSA (2015-2018) because it included the validated 3-item University of California, Los Angeles (UCLA) loneliness scale [22]. Our sample is from both the Tracking cohort version 2.1 (n=17,051) and Comprehensive cohort version 3.0 (n=27,765), follow-up 1 survey (n=44,816). We then excluded 403 respondents who did not answer the 3-item UCLA loneliness scale or had other missing responses. This resulted in 0.90% (403/44,816) missing data. Given the small percentage of missing data, we used listwise deletion to manage missing responses. The final sample was n=44,413.

### Loneliness

Our primary independent variable of loneliness was measured using the 3-item UCLA loneliness scale [23]; one of most widely used scales to assess loneliness. The items ask, 1) How often do you feel left out?; 2) How often do you feel isolated from others?; and 3) How often do you feel that you lack companionship?” Items are scored with a Likert scale response category (Hardly ever, Some of the time, Often). Overall scores range from 3 to 9, with higher scores indicating greater perceived loneliness. Exploratory and confirmatory factor analyses have demonstrated robust reliability and concurrent and discriminant validity when used in large, population-based samples [22]. To create a dichotomous measure (lonely versus not lonely), we identified individuals in the top quintile of our sample compared to remaining individuals. This approach has been used in other studies [24]. Based on the distribution of our sample, the score for lonely was ≥5 out of 9.

### Self-Reported History of Emergency Department Use

The outcome was assessed using the following question: “*Have you been seen in an emergency department during the past 12 months?”.* It was measured as any (yes/no) self-reported ED use in the 12 months prior to the survey date.

### Sex and Gender

Our study has the unique opportunity to examine the relationship between loneliness, gender, and ED visits. The CLSA has both a measure of sex and gender. Sex is measured as the respondent’s sex at birth (Male, Female). Gender is measured as the respondent’s current gender identity (male, female, transgender man/transman, transgender woman/transwoman, genderqueer, other). We examined the overlap between each sex and gender variable and found high concordance between the two measures (see Supplementary Table 1) so we chose to include only gender in our analyses. We chose to focus on gender identity, rather than sex at birth, because other research has shown that gendered attitudes, self-perception, and social factors are related to loneliness [4, 25] and our interest was not biological differences. We ultimately created a variable based on the respondent’s current gender identity, as one of: men (includes transmen), women (includes transwomen), and gender diverse (gender queer, other, do not know). These three categories are consistent with others, including Statistics Canada’s gender classification [26].

### Sociodemographic and health variables

Other variables included in the analysis were selected based on their relationship to loneliness and ED visits in community-dwelling older adults, as shown in prior research [4, 11, 12, 27–30]. All variables in our analysis are self-reported. We examined respondent demographics including age (<65 years, 65+ years), education (less than university degree, university degree or higher), ethnicity (White, all else), household income (<$20,000, $20,000-<$50,000, $50,000+), marital status (single, never married, divorced/separated, married/common-law, widowed), living arrangement (live alone=yes/no), and geographic location (urban, rural).

Health related variables included number of chronic conditions. The number of potential chronic illnesses ranged from 1 to 18, the median was 2 and the 75^th^ percentile (or 3^rd^ quartile) value was 3. We used the 75^th^ percentile as the cut off to create a dichotomous variable (1-3 vs 4+). Functional impairment was measured using the 5-point scale from the Older Americans’ Resources and Services (OARS) Multidimensional Functional Assessment Questionnaire, which ranges from 2 (Excellent/Good) to 6 (Total Impairment). The CLSA modified this scale so that the range is 1 to 5, and the categories are (none, mild/moderate/severe/total impairment for activities of daily living). Higher values indicate greater impairment [31]. Self-rated mental health was assessed through the following question: “*In general, would you say your mental health is excellent, very good, good, fair or poor?”* (recategorized as: poor, fair/good/very good/excellent), and diagnosis of anxiety (yes, no). The number of depressive symptoms over the past week was measured using the Center for Epidemiologic Studies Depression Scale (CES-D) [32]. The 10-item CES-D scale generates a score between 0 and 30 with higher scores indicating a greater number of depressive symptoms. The 10-item scale includes an item that asks whether the respondent has felt lonely. We removed this item and created a revised scale to avoid overlap with our loneliness measure, which is consistent with other research [24, 33]. We adopted a cut off of <10 and 10+ [32]. Other health service use was measured by asking if the respondent had visited their family doctor in the last 12 months (yes/no).

We also assessed factors related to social isolation, which is often measured by the number of contacts, living arrangements (living alone versus with others), and participation in social activities [34, 35]. We identified whether respondents had seen any of the following social contacts within the last 6 months: children, siblings, other relatives, close friends, neighbors. For social participation, we assessed whether respondents had participated in any of the following activities within the last 6 months: family or friendship activities outside the household, church or religious activities, sports or physical activities, educational and cultural activities, service club or fraternal organizational activities. For each social contact and social participation, respondents were scored from 0-5. We categorized each social contact and activity participation to the following: 0-1=low contact/participation, 2-3=moderate contact/participation, 4-5=high contact/participation.

### Analysis

We calculated descriptive statistics using the unweighted data for all variables. We present frequencies and percentages for categorical variables and median and interquartile range (IQR) for continuous variables. We calculated standardized differences in proportions for all variables by loneliness (lonely/not lonely). Standardized difference scores are indexes which measure the effect size between two groups and are preferred in large samples [36]. There is no agreed upon threshold for standardized differences that indicate imbalance however, it is suggested that a difference in proportion of less than 10% (0.1) indicates little difference [36]. We presented our descriptive findings in our pooled sample by lonely/not lonely and by gender (men, women, gender diverse). We also examined our findings by age (<65, 65+) (Supplementary Tables 2-3). We did not report any cell sizes smaller than six and our tables have the following notation (0-5) when this is the case. This reporting practice is consistent with guidance to limit privacy risks when groups or attributes (i.e., gender identity) have a small denominator or the type of data being reported is unique and might carry a higher risk of identification [37].

We used multivariable logistic regression analysis to examine the association between loneliness and ED visit in the previous 12 months, controlling for other demographic, health, and social factors. We used the analytic weights provided by CLSA for the logistic regression models. Unadjusted odds ratios (uOR) and adjusted odds ratios (aOR) are presented. Multivariable analyses were run in the full sample and then disaggregated by gender (men, women) and age (<65, 65+). Due to the small number of gender diverse respondents, the logistic regression in this analysis adjusted only for age. Statistical software used in the analyses were IBMSPSS version 26 and SAS 9.4. We received research ethics approval from the University of Alberta (Pro00100416). We followed the Strengthening the Reporting of Observational Studies in Epidemiology (STROBE) guidelines for reporting observational cohort studies.

## Results

Overall, our sample included 44,413 participants. 23.1% (n=10263) were identified as lonely (Table 1). Gender diverse respondents had the highest percentage identified as lonely (28.2%, n=11), followed by women (25.4%, n=5765), and men (20.6%, n=4483). Our total sample was predominantly White (96.3%, n=42884) and lived in an urban area (89.6%, n=39870). Over 70% (70.7%, n=24224) who were not lonely had a household income of $50,000+ compared to 51.8% (n=5314) who were lonely. Nearly 20% (19.8%, n=8821) of our total sample reported having 4+ chronic conditions. When we examined chronic conditions and loneliness, 29.3% (n=3010) lonely of respondents had 4+ chronic conditions compared to 17% (n=5811) who were not lonely. Lonely respondents were more likely to report functional impairment (22.1%, n=2263) compared to not lonely (11.2%, n=3839). Less than a quarter of our sample had been to the ED in the prior year (22.5%, n=10009). Of those that were lonely, 27.2% (n=2793) had been to the ED in the prior year compared to 21.1% (n=7216) who were not lonely. Over 90% of respondents had been to a family doctor in the last 12 months (90.9%, n=40471), this differed only slightly when examined by lonely (91.4%, n=9378) and not lonely (90.9%, n=31093).

**Table 1:** Demographic characteristics, health, social, and health service use in 44,518 Canadian Longitudinal Study on Aging Tracking and Comprehensive Follow-up 1 survey respondents from 2015-2018 by loneliness (lonely, not lonely) and gender (men, women, gender diverse)

Variables	Total	Lonely (n=44,518)		Std. difference	Men (n=21,722)		Women (n=22,740)		Gender Diverse (n=39)	
					Lonely		Lonely		Lonely	
		No	Yes		No	Yes	No	Yes	No	Yes
	N (%)	N (%)	N (%)		N (%)	N (%)	N (%)	N (%)	N (%)	N (%)
Total	44518 (100)	34255 (76.9)	10263 (23.1)		17239 (79.4)	4483 (20.6)	16975 (74.6)	5765 (25.4)	28 (71.8)	11 (28.2)
**ED visit**										
Yes	10009 (22.5)	7216 (21.1)	2793 (27.2)	0.14	3614 (21)	1180 (26.3)	3595 (21.2)	1606 (27.9)	0-5	6 (54.5)
No	34385 (77.2)	26956 (78.7)	7429 (72.4)	0.15	13590 (78.8)	3281 (73.2)	13333 (78.5)	4140 (71.8)	23 (82.1)	0-5
**Age (years)**										
<65	21201 (47.6)	16253 (47.4)	4948 (48.2)	0.02	7922 (46)	2187 (48.8)	8314 (49)	2751 (47.7)	15 (53.6)	7 (63.6)
65+	23317 (52.4)	18002 (52.6)	5315 (51.8)	0.02	9317 (54)	2296 (51.2)	8661 (51)	3014 (52.3)	13 (46.4)	0-5
**Education**										
Less than university	22882 (51.4)	17320 (50.6)	5562 (54.2)	0.07	8038 (46.6)	2242 (50)	9265 (54.6)	3317 (57.5)	9 (32.1)	0-5
University or higher	18965 (42.6)	15106 (44.1)	3859 (37.6)	0.13	8310 (48.2)	1907 (42.5)	6774 (39.9)	1945 (33.7)	18 (64.3)	0-5
**Ethnicity**										
All else	1634 (3.7)	1150 (3.4)	484 (4.7)	0.07	660 (3.8)	253 (5.6)	490 (2.9)	231 (4)	0-5	0-5
White	42884 (96.3)	33105 (96.6)	9779 (95.3)	0.07	16579 (96.2)	4230 (94.4)	16485 (97.1)	5534 (96)	28 (100)	11 (100)
**Geographic region**										
Rural	4648 (10.4)	3633 (10.6)	1015 (9.9)	0.02	1841 (10.7)	468 (10.4)	1783 (10.5)	546 (9.5)	0-5	0-5
Urban	39870 (89.6)	30622 (89.4)	9248 (90.1)	0.02	15398 (89.3)	4015 (89.6)	15192 (89.5)	5219 (90.5)	23 (82.1)	11 (100)
**Income**										
<$20,000	2080 (4.7)	1100 (3.2)	980 (9.5)	0.26	339 (2)	334 (7.5)	757 (4.5)	640 (11.1)	0-5	0-5
$20,000-<$50,000	9928 (22.3)	6826 (19.9)	3102 (30.2)	0.24	2802 (16.3)	1183 (26.4)	4013 (23.6)	1916 (33.2)	9 (32.1)	0-5
$50,000+	29538 (66.4)	24224 (70.7)	5314 (51.8)	0.40	13350 (77.4)	2667 (59.5)	10854 (63.9)	2642 (45.8)	14 (50)	0-5
**Marital status**										
Single, never married	3855 (8.7)	2390 (7)	1465 (14.3)	0.24	1024 (5.9)	703 (15.7)	1356 (8)	759 (13.2)	0-5	0-5
Divorced/separated	4884 (11)	3086 (9)	1798 (17.5)	0.25	698 (4)	552 (12.3)	2385 (14.1)	1242 (21.5)	0-5	0-5
Married/common law	30354 (68.2)	25435 (74.3)	4919 (47.9)	0.56	14444 (83.8)	2463 (54.9)	10966 (64.6)	2450 (42.5)	18 (64.3)	0-5
Widowed	5403 (12.1)	3326 (9.7)	2077 (20.2)	0.30	1066 (6.2)	764 (17)	2257 (13.3)	1311 (22.7)	0-5	0-5
**Living alone**										
No	33244 (74.7)	27287 (79.7)	5957 (58)	0.48	14910 (86.5)	2794 (62.3)	12350 (72.8)	3158 (54.8)	20 (71.4)	0-5
Yes	11274 (25.3)	6968 (20.3)	4306 (42)	0.48	2329 (13.5)	1689 (37.7)	4625 (27.2)	2607 (45.2)	8 (28.6)	9 (81.8)
**Number of chronic conditions**										
<4	35697 (80.2)	28444 (83)	7253 (70.7)	0.30	15305 (88.8)	3562 (79.5)	13106 (77.2)	3683 (63.9)	24 (85.7)	6 (54.5)
4+	8821 (19.8)	5811 (17)	3010 (29.3)	0.30	1934 (11.2)	921 (20.5)	3869 (22.8)	2082 (36.1)	0-5	0-5
**Functional impairment**										
None	37074 (83.3)	29395 (85.8)	7679 (74.8)	0.28	15835 (91.9)	3743 (83.5)	13528 (79.7)	3927 (68.1)	25 (89.3)	6 (54.5)
Mild/moderate/severe/total	6102 (13.7)	3839 (11.2)	2263 (22.1)	0.29	1223 (7.1)	688 (15.3)	2609 (15.4)	1570 (27.2)	0-5	0-5
**Self-rated mental health**										
Poor	389 (0.9)	118 (0.3)	271 (2.6)	0.19	57 (0.3)	129 (2.9)	61 (0.4)	142 (2.5)	0-5	0-5
Fair/Good/Very Good/Excellent	44066 (99)	34101 (99.6)	9965 (97.1)	0.19	17166 (99.6)	4344 (96.9)	16895 (99.5)	5608 (97.3)	28 (100)	9 (81.8)
**Number of depressive symptoms**										
Median (Q1-Q3)	19 (17-21)	19 (17-21)	17 (15-20)	0.03	19 (17-21)	19 (17-21)	18 (16-20)	18 (16-20)	19 (16-21)	19 (16-20)
<10	789 (1.8)	329 (1)	460 (4.5)	-0.22	112 (0.6)	156 (3.5)	217 (1.3)	303 (5.3)	0-5	0-5
10+	42658 (95.8)	33132 (96.7)	9526 (92.8)	0.18	16726 (97)	4203 (93.8)	16367 (96.4)	5309 (92.1)	27 (96.4)	10 (90.9)
**Number of social contacts**										
Median (Q1-Q3)	3 (3-4)	4 (3-4)	3 (2-4)		3 (2-4)	3 (2-4)	4(3-4)	4 (3-4)	3 (2-3)	3 (2-3)
High contact (4-5)	22124 (49.7)	17899 (52.3)	4225 (41.2)	0.22	8530 (49.5)	1674 (37.3)	9358 (55.1)	2547 (44.2)	7 (25)	0-5
Moderate contact (2-3)	18793 (42.2)	13977 (40.8)	4816 (46.9)	0.12	7331 (42.5)	2180 (48.6)	6620 (39)	2630 (45.6)	20 (71.4)	0-5
Low contact (0-1)	3600 (8.1)	2378 (6.9)	1222 (11.9)	0.17	1378 (8)	629 (14)	996 (5.9)	588 (10.2)	0-5	0-5
**Number of social activities**										
Median (Q1-Q3)	3 (2-3)	3 (2-3)	2 (1-3)		3 (2-3)	3 (2-3)	3 (2-3)	3 (2-3)	2 (1-3)	2 (1-3)
High participation (4-5)	9223 (20.7)	7567 (22.1)	1656 (16.1)	0.15	3495 (20.3)	647 (14.4)	4063 (23.9)	1009 (17.5)	7 (25)	0-5
Moderate participation (2-3)	26852 (60.3)	21084 (61.6)	5768 (56.2)	0.11	10617 (61.6)	2512 (56)	10446 (61.5)	3251 (56.4)	15 (53.6)	0-5
Low participation (0-1)	7814 (17.6)	5158 (15.1)	2656 (25.9)	0.27	2935 (17)	1234 (27.5)	2213 (13)	1412 (24.5)	6 (21.4)	9 (81.8)
**Anxiety**										
No	39462 (88.6)	31060 (90.7)	8402 (81.9)	0.26	15939 (92.5)	3780 (84.3)	15082 (88.8)	4610 (80)	27 (96.4)	9 (81.8)
Yes	3980 (8.9)	2401 (7)	1579 (15.4)	0.27	893 (5.2)	578 (12.9)	1508 (8.9)	998 (17.3)	0-5	0-5
**Family doctor in last 12 months**										
No	3939 (8.8)	3086 (9)	853 (8.3)	0.02	1694 (9.8)	440 (9.8)	1389 (8.2)	413 (7.2)	0-5	0-5
Yes	40471 (90.9)	31093 (90.8)	9378 (91.4)	0.02	15515 (90)	4022 (89.7)	15541 (91.6)	5341 (92.6)	26 (92.9)	11 (100)

Differences in the characteristics of those who were lonely emerged by gender (Table 1). Men who were lonely were more likely to have a university degree or higher (42.5%, n=1907) compared to women who were lonely (33.7%, n=1945). Women who were lonely had lower household income (45.8%, n=2642) than men (59.5%, n=2667) and more frequently lived alone (45.2%, n=2607 versus 37.7%, n=1689). Over 80% (81.8%, n=9) of lonely gender diverse respondents lived alone.

We examined the prevalence of factors related to social isolation, including living alone, number of social contacts, and social participation. Over one-quarter (25.3%, n=11274) of our sample lived alone, which differed for those who were lonely (42%, n=4306) and not lonely (20.3%, n=6968). Most of the sample had a high social contact (49.7%, n=22124), and moderate participation in social activities (60.3%, n=26852). A higher proportion of lonely individuals reported low social participation (25.9%, n=2656) compared to not lonely (15.1%, n=5158). A similar pattern was observed between lonely/not lonely and low social contact.

### Loneliness and ED visits

Compared to those who were not lonely, those who were lonely had higher odds of an ED visit (aOR: 1.13, 95% CI: 1.05-1.21), adjusted for demographic, health, and social factors (Table 2). In our full sample (not stratified by gender), women had lower odds of ED visit (aOR: 0.86, 95% CI: 0.81-0.92) compared to men. We examined the same predictors in separate logistic regressions for men and women (Table 3). Loneliness was associated with ED visits in women (aOR: 1.15, 95% CI: 1.05-1.25) more so than in men (aOR: 1.10, 95% CI: 0.99-1.22). We ran the logistic regression in the gender-diverse group that included only loneliness and age as the covariates (Table 4). In this analysis, loneliness was significantly associated with ED visits among gender-diverse respondents (aOR: 1.43, 95% CI: 1.33-1.54). We also tested the association by age groups (<65, 65+) and found that the association between loneliness and ED visits persisted in those aged less than 65 years of age (aOR: 1.11, 95% CI: 1.01-1.22) and those 65 years of age and older (aOR: 1.14, 95% CI: 1.04-1.25) (Supplementary Tables 4-5).

**Table 2:** Logistic regression model results to test associations between loneliness and ED visit in previous 12 months in 44,518 Canadian Longitudinal Study on Aging Tracking and Comprehensive Follow-up 1 (2015-2018) survey respondents

Variables	Odds Ratio (95% CI) *N*=44,518	
	Unadjusted	Adjusted (weighted)^a^
**Lonely** (ref: not lonely)	1.41 (1.34-1.48)	1.13 (1.05-1.21)

^a^Adjusted for education, ethnicity, age, geographic region, gender, income, marital status, living alone, chronic conditions, functional impairment, self-rated mental health, depressive symptoms, anxiety, social contacts, social activities, family physician visit.

**Table 3:** Logistic regression model results by gender (men, women) to test associations between loneliness and ED visit in Canadian Longitudinal Study on Aging Tracking and Comprehensive Follow-up 1 (2015-2018) survey

	Men (*n*=21,722) Odds Ratio (95% CI)		Women (*n*=22,722) Odds Ratio (95% CI)	
	Unadjusted	Adjusted (weighted)^a^	Unadjusted	Adjusted (weighted)^a^
**Lonely** (ref: not lonely)	1.35 (1.25-1.46)	1.10 (0.99-1.22)	1.44 (1.34-1.54)	1.15 (1.05-1.25)

^a^Adjusted for education, ethnicity, age, geographic region, income, marital status, living alone, chronic conditions, functional impairment, self-rated mental health, depressive symptoms, anxiety, social contacts, social activities, family physician visit

**Table 4:** Logistic regression model results to test associations between loneliness and ED visit among gender diverse respondents in Canadian Longitudinal Study on Aging Tracking and Comprehensive Follow-up 1 (2015-2018) survey

	Gender diverse (*n*=39) Odds Ratio (95% CI)	
	Unadjusted	Adjusted (weighted)
Lonely (ref: not lonely)	5.52 (1.19-25.52)	1.43 (1.33-1.54)
Age 65+ (ref: <65)	1.11 (0.27-4.52)	1.28 (1.19-1.36)

## Discussion

In a population-based sample of community-dwelling older adults, 23.1% were lonely. Overall, those who were gender diverse, which was the smallest group, had the highest frequency of loneliness, followed by women and men, respectively. We found that loneliness was associated with higher odds of ED visit, controlling for other demographic, health, and social factors. In our gender-stratified analysis, loneliness was significantly associated with previous ED visits in women but less so in men; loneliness was most strongly associated with ED visits among those identifying as gender diverse, however, the small sample size prevented adjustment for several potentially confounding variables. Our age-stratified regression analysis (<65, 65+) also found that loneliness was significantly associated with previous ED visits, adjusted for other demographic, health, and social factors.

Our findings add to the growing body of research examining social relationships and health service use. While the literature results vary in the consensus on the relationship between loneliness and health service use, our findings support the association between loneliness and ED visits. We know that loneliness is associated with various health conditions and morbidity [1, 38], which may lead to increased ED use; however, our models controlled for various health conditions and functional impairments. This suggests that there is some other mechanism leading to an association between loneliness and ED visits that is independent of major health conditions. Loneliness is the feeling that results from a discrepancy in the desired quantity and quality of social relationships [39]. This discrepancy is physiologically distressing and can predict subsequent functional decline and risk of morbidity and mortalit y [1, 40]. Hawkley at a l [39]. suggest that the pain of loneliness can motivate the formation or reconnection of social relationships. Some studies suggest that lonely people seek out medical care because they are looking for social interaction [10, 41]. Loneliness is also related to other aspects of social functioning including the ability to harness social support and social resources. Cacioppo et al . [42] considered the evolutionary mechanisms of loneliness and suggested that when the social pain of loneliness is activated it can set off innate survival mechanisms like hypervigilance. Hypervigilance to external cues can set off a cascade of actions which limit the individual’s ability to self-regulate their behaviours, to carry out maintenance tasks including health-promoting behaviours, and to identify others who could assist with those tasks [42]. It may be that those lonely individuals end up in ED because these hypervigilance cues inhibit their ability to manage their health at home and engage in preventative care tasks that help keep them from the ED. Further, those who are lonely may lack a robust social support system which may also contribute to a diminished capacity to oversee preventative care tasks, leading to ED visits. Using the CLSA data, we were only able to assess whether respondents had been to ED in the past 12 months and not the frequency of ED visits, the reason for the visit, or if they had any repeated visits. Our future work aims to use administrative health data to better understand how loneliness influences both frequency and repeat ED visits to understand how loneliness may set off different trajectories of care use.

Our gender-stratified analysis showed slight differences in the association between loneliness and odds of ED visit between women and men. Women had greater odds of an ED visit given loneliness compared to men. Similar results were found in a recent study using data from the Irish Longitudinal Study on Ageing that examined loneliness in community-dwelling adults and its association with self-reported general practitioner (GP) and ED visits in the last 12 months [43]. Loneliness in men did not influence ED or GP visits but women who reported loneliness had elevated risk of ED visit. Our observed differences between men and women may be due to the social stigma of loneliness which can effect the respondents likelihood of recognizing or disclosing loneliness [44]. Males may be less likely to self-disclose loneliness due to stereotypes of masculinity and the stigma of emotional expression [4]. Our preliminary findings in the gender-diverse respondents suggest that loneliness is also associated with ED use in this group. Research in Australia examined loneliness severity in lesbian, gay, bisexual, transgender, queer, intersex, asexual, and other sexual orientation and gender identity diverse (LGBTQIA+) communities. They found that LGBTQIA+ adults had higher levels of loneliness than non- LGBTQIA+ adults [45]. Sanchez et al. [46] examined ED utilization in a sample of 360 lesbian, gay, and bisexual individuals and found that over the previous 12 months, 25.3% had at least one ED visit and 16% had two or more visits. This study did not examine participant social connections such as loneliness. These studies highlight the emerging area of research examining the intersections of loneliness and health service use in gender diverse communities.

Our sample did not include a robust sample of persons that have been historically excluded and underrepresented in research such as racial and ethnic minorities, socioeconomically disadvantaged populations, rural populations, and sexual and gender minorities [47, 48]. Our sample had a small proportion of people who were non-White which is problematic because other research has found that sociodemographic variables like race are predictors of ED visits [17, 27]. Although we used weighted data from a large population-based survey, the respondents are relatively homogenous, they are predominantly White, live in urban areas, are highly educated and have relatively high household incomes. The CLSA weights reflect the Canadian population on many variables and as the study continues these weights are designed to best reflect the true underlying population, but there are limitations. The format and inclusion/exclusion criteria of the CLSA data collection includes telephone interviews and in-person interviews at a regional assessment centre which limits participation from those who do not have access to a phone, transportation to a data collection centre, or who cannot complete the survey in English or French. Similarly, we were also unable to fully examine gender diverse respondents due to a small sample size. The inclusion of gender identity categories in the CLSA is an important and essential step to reflect the realities of respondents; however, it is not sufficient. Integrating a question about gender requires that sampling be robust so that there are sufficient numbers of respondents from these groups to ensure that the data they provide can be meaningfully included in quantitative analysis and not automatically excluded due to sample size issues. Efforts must be made to enhance our ability to understand the aging experience for a more diverse group of Canadians. If we are to develop testable hypotheses and posit how a population uses (or does not use) a health service, it is imperative that we include a truly representative sample of Canadians who engage with the health system.

## Limitations

Although we examined a large population-based sample of community-dwelling adults middle-aged and older adults, our study has limitations. It is difficult to compare our findings to other studies examining ED utilization, because they often vary in outcome measurement (dichotomous, count), time periods (6 weeks, 3 months, 1 year), and sources (self-report, administrative health data, chart review) [17]. We only had one ED use item (yes/no) that was self-report and at this time cannot link data from the CLSA to ED use information from administrative health data. Studies comparing health service utilization between self-report versus administrative health data often show poor to fair agreement between the two measures but comparable predictive accuracy in subsequent models [49, 50]. Future work is needed to link these CLSA self-report data on prior ED use to available administrative health data. Our data are cross-sectional so we cannot infer temporality and how loneliness may influence future ED use. Data on frequency of visits, re-admissions to ED, and/or other outcomes (such as discharge home vs admission) may provide additional information on use patterns between older adults who are and are not lonely [18]. Similarly, we do not know the reason for ED visit (e.g., self-care problems, falls) and this information may be related to loneliness. As we have noted in our discussion, we were unable to interpret much of the data on gender diverse respondents given the small sample size.

## Conclusion

Loneliness was prevalent in our sample and our findings suggest that there are differences in the prevalence across genders. Loneliness is associated with previous ED visits, controlling for numerous demographic, health, and social factors. This highlights an important area of inquiry both for researchers and perhaps more importantly for practitioners and health systems, if loneliness influences health system use then how can it be identified and measured in our health system? Systematic measurement is crucial for early intervention. Finally, our findings from gender-diverse respondents suggest this group may differ from others in their loneliness and odds of ED visit. This preliminary work highlights an important area of future study and consideration in intervention development.

## Supplementary Information

**Additional file 1.**

## Data Availability

Data are available from the Canadian Longitudinal Study on Aging (www.clsa-elcv.ca) for researchers who meet the criteria for access to de-identified CLSA data.

## Abbreviations

CLSA: Canadian Longitudinal Study on Aging
ED: Emergency department
