# Supplementary material for: Examining the association between loneliness and emergency department visits using Canadian Longitudinal Study of Aging (CLSA) data: a retrospective cross-sectional study
Source: BMC Geriatr. 2022 Jan 22;22:69. doi: 10.1186/s12877-022-02763-8 (PMC8783523; doi:10.1186/s12877-022-02763-8)
Supplement: Supplementary file 1 — Additional file 1. [file 12877_2022_2763_MOESM1_ESM.docx]

Supplementary Table 1. Sex at birth and current gender identity

| **Sex at birth** | **Gender identity** | | | | | | | |  |
| --- | --- | --- | --- | --- | --- | --- | --- | --- | --- |
|  | Male | Female | Transman | Transwoman | Genderqueer | Other | Don’t know/No answer | Refused | Total |
| Male | 21826 | 28 | 3 | 4 | 3 | 10 | 4 | 3 | 21881 |
| Female | 23 | 22863 | 2 | 3 | 2 | 11 | 5 | 2 | 22911 |
| Don’t know/No answer | 1 | 2 | 0 | 0 | 0 | 0 | 3 | 0 | 6 |
| Refused | 3 | 2 | 0 | 0 | 0 | 1 | 0 | 12 | 18 |
| Total | 21853 | 22895 | 5 | 7 | 5 | 22 | 12 | 17 | 44816 |

Supplementary Table 2. Demographic characteristics, health, social, and health service use in 21,201 Canadian Longitudinal Study on Aging Tracking and Comprehensive Follow-up 1 survey respondents aged less than 65 years from 2015-2018 by loneliness (lonely, not lonely) and gender (men, women, gender diverse)

| **Variables** | **Total** | **Lonely (n=21,201)** | | **Std. difference** | **Men (n=11,109)** | | **Women (n=11,065)** | | **Gender Diverse (n=22)** | |
| --- | --- | --- | --- | --- | --- | --- | --- | --- | --- | --- |
|  |  |  |  |  | **Lonely** | | **Lonely** | | **Lonely** | |
|  |  | **No** | **Yes** |  | **No** | **Yes** | **No** | **Yes** | **No** | **Yes** |
|  | **N (%)** | **N (%)** | **N (%)** |  | **N (%)** | **N (%)** | **N (%)** | **N (%)** | **N (%)** | **N (%)** |
| **Total** | 21201 | 16253 | 4948 |  | 7922 | 2187 | 8314 | 2751 | 15 | 7 |
| **ED visit** |  |  |  |  |  |  |  |  |  |  |
| Yes | 4254(20.1) | 3025(18.6) | 1229(24.8) | -0.15 | 1447(18.3) | 508(23.2) | 1576(19) | 716(26) | 2(13.3) | 4(57.1) |
| No | 16901(79.7) | 13196(81.2) | 3705(74.9) | 0.15 | 6461(81.6) | 1672(76.5) | 6720(80.8) | 2028(73.7) | 13(86.7) | 3(42.9) |
| **Education** |  |  |  |  |  |  |  |  |  |  |
| Less than university | 10969(51.7) | 8225(50.6) | 2744(55.5) | -0.10 | 3855(48.7) | 1158(52.9) | 4364(52.5) | 1584(57.6) | 4(26.7) | 2(28.6) |
| University or higher | 9590(45.2) | 7611(46.8) | 1979(40) | 0.14 | 3845(48.5) | 919(42) | 3755(45.2) | 1054(38.3) | 11(73.3) | 4(57.1) |
| **Ethnicity** |  |  |  |  |  |  |  |  |  |  |
| All else | 949(4.5) | 666(4.1) | 283(5.7) | -0.08 | 357(4.5) | 147(6.7) | 309(3.7) | 136(4.9) | 0(0) | 0(0) |
| White | 20252(95.5) | 15587(95.9) | 4665(94.3) | 0.08 | 7565(95.5) | 2040(93.3) | 8005(96.3) | 2615(95.1) | 15(100) | 7(100) |
| **Geographic region** |  |  |  |  |  |  |  |  |  |  |
| Rural | 2317(10.9) | 1818(11.2) | 499(10.1) | 0.04 | 859(10.8) | 219(10) | 956(11.5) | 279(10.1) | 2(13.3) | 0(0) |
| Urban | 18884(89.1) | 14435(88.8) | 4449(89.9) | -0.04 | 7063(89.2) | 1968(90) | 7358(88.5) | 2472(89.9) | 13(86.7) | 7(100) |
| **Income** |  |  |  |  |  |  |  |  |  |  |
| <$20,000 | 771(3.6) | 323(2) | 448(9.1) | -0.31 | 129(1.6) | 175(8) | 192(2.3) | 270(9.8) | 2(13.3) | 2(28.6) |
| $20,000-<$50,000 | 2831(13.4) | 1781(11) | 1050(21.2) | -0.28 | 676(8.5) | 405(18.5) | 1102(13.3) | 642(23.3) | 2(13.3) | 3(42.9) |
| $50,000+ | 16653(78.5) | 13508(83.1) | 3145(63.6) | 0.45 | 6884(86.9) | 1483(67.8) | 6614(79.6) | 1659(60.3) | 9(60) | 1(14.3) |
| **Marital status** |  |  |  |  |  |  |  |  |  |  |
| Single, never married | 2313(10.9) | 1362(8.4) | 951(19.2) | -0.32 | 605(7.6) | 465(21.3) | 754(9.1) | 483(17.6) | 3(20) | 2(28.6) |
| Divorced/separated | 558(2.6) | 293(1.8) | 265(5.4) | -0.19 | 65(0.8) | 80(3.7) | 228(2.7) | 184(6.7) | 0(0) | 1(14.3) |
| Married/common law | 15798(74.5) | 13128(80.8) | 2670(54) | 0.60 | 6757(85.3) | 1239(56.7) | 6358(76.5) | 1427(51.9) | 11(73.3) | 2(28.6) |
| Widowed | 2524(11.9) | 1464(9) | 1060(21.4) | -0.35 | 494(6.2) | 402(18.4) | 969(11.7) | 656(23.8) | 1(6.7) | 2(28.6) |
| **Living alone** |  |  |  |  |  |  |  |  |  |  |
| No | 17572(82.9) | 14216(87.5) | 3356(67.8) | 0.49 | 7110(89.8) | 1486(67.9) | 7092(85.3) | 1867(67.9) | 12(80) | 1(14.3) |
| Yes | 3629(17.1) | 2037(12.5) | 1592(32.2) | -0.49 | 812(10.2) | 701(32.1) | 1222(14.7) | 884(32.1) | 3(20) | 6(85.7) |
| **Number of chronic conditions** |  |  |  |  |  |  |  |  |  |  |
| <4 | 18416(86.9) | 14544(89.5) | 3872(78.3) | 0.31 | 7438(93.9) | 1873(85.6) | 7092(85.3) | 1992(72.4) | 12(80) | 5(71.4) |
| 4+ | 2785(13.1) | 1709(10.5) | 1076(21.7) | -0.31 | 484(6.1) | 314(14.4) | 1222(14.7) | 759(27.6) | 3(20) | 2(28.6) |
| **Functional impairment** |  |  |  |  |  |  |  |  |  |  |
| None | 19028(89.8) | 14918(91.8) | 4110(83.1) | 0.27 | 7621(96.2) | 1959(89.6) | 7281(87.6) | 2144(77.9) | 15(100) | 5(71.4) |
| Mild/moderate/severe/total | 1517(7.2) | 834(5.1) | 683(13.8) | -0.30 | 222(2.8) | 207(9.5) | 611(7.3) | 474(17.2) | 0(0) | 1(14.3) |
| **Self-rated mental health** |  |  |  |  |  |  |  |  |  |  |
| Poor | 248(1.2) | 68(0.4) | 180(3.6) | -0.23 | 33(0.4) | 86(3.9) | 35(0.4) | 94(3.4) | 0(0) | 0(0) |
| Fair/Good/Very Good/Excellent | 20936(98.8) | 16176(99.5) | 4760(96.2) | 0.23 | 7885(99.5) | 2097(95.9) | 8274(99.5) | 2654(96.5) | 15(100) | 6(85.7) |
| **Number of depressive symptoms** |  |  |  |  |  |  |  |  |  |  |
| Median (Q1-Q3) | 19(17-21) | 19(17-21) | 18(15-20) |  | 20(18-21) | 18(18-22) | 19(17-20) | 17(14-19) | 20(19-21) | 18(16-21) |
| <10 | 418(2) | 159(1) | 259(5.2) | -0.25 | 45(0.6) | 92(4.2) | 114(1.4) | 167(6.1) | 0(0) | 0(0) |
| 10+ | 20318(95.8) | 15751(96.9) | 4567(92.3) | 0.21 | 7695(97.1) | 2046(93.6) | 8039(96.7) | 2511(91.3) | 15(100) | 7(100) |
| **Number of social contacts** |  |  |  |  |  |  |  |  |  |  |
| Median (Q1-Q3) | 4(3-4) | 4(3-4) | 3(2-4) |  | 4(3-4) | 3(2-4) | 4(3-4) | 3(2-4) | 2(2-3) | 1(1-3) |
| High contact (4-5) | 10845(51.2) | 8845(54.4) | 2000(40.4) | 0.28 | 4051(51.1) | 802(36.7) | 4790(57.6) | 1196(43.5) | 4(26.7) | 1(14.3) |
| Moderate contact (2-3) | 8733(41.2) | 6392(39.3) | 2341(47.3) | -0.16 | 3285(41.5) | 1070(48.9) | 3096(37.2) | 1267(46.1) | 10(66.7) | 2(28.6) |
| Low contact (0-1) | 1623(7.7) | 1016(6.3) | 607(12.3) | -0.21 | 586(7.4) | 315(14.4) | 428(5.1) | 288(10.5) | 1(6.7) | 4(57.1) |
| **Number of social activities** |  |  |  |  |  |  |  |  |  |  |
| Median (Q1-Q3) | 3(2-3) | 3(2-3) | 2(1-3) |  | 3(2-3) | 2(1-3) | 3(2-3) | 2(1-3) | 3(2-4) | 1(0-1) |
| High participation (4-5) | 3618(17.1) | 2984(18.4) | 634(12.8) | 0.15 | 1350(17) | 264(12.1) | 1630(19.6) | 370(13.4) | 4(26.7) | 0(0) |
| Moderate participation (2-3) | 13694(64.6) | 10769(66.3) | 2925(59.1) | 0.15 | 5214(65.8) | 1280(58.5) | 5547(66.7) | 1642(59.7) | 8(53.3) | 1(14.3) |
| Low participation (0-1) | 3628(17.1) | 2324(14.3) | 1304(26.4) | -0.30 | 1282(16.2) | 606(27.7) | 1037(12.5) | 691(25.1) | 3(20) | 6(85.7) |
| **Anxiety** |  |  |  |  |  |  |  |  |  |  |
| No | 18418(86.9) | 14542(89.5) | 3876(78.3) | 0.31 | 7229(91.3) | 1787(81.7) | 7296(87.8) | 2081(75.6) | 15(100) | 6(85.7) |
| Yes | 2307(10.9) | 1365(8.4) | 942(19) | -0.31 | 510(6.4) | 349(16) | 855(10.3) | 591(21.5) | 0(0) | 1(14.3) |
| **Family doctor in last 12 months** |  |  |  |  |  |  |  |  |  |  |
| No | 2661(12.6) | 2107(13) | 554(11.2) | 0.05 | 1176(14.8) | 307(14) | 930(11.2) | 247(9) | 1(6.7) | 0(0) |
| Yes | 18486(87.2) | 14107(86.8) | 4379(88.5) | -0.05 | 6731(85) | 1871(85.6) | 7360(88.5) | 2498(90.8) | 14(93.3) | 7(100) |

Supplementary Table 3 Demographic characteristics, health, social, and health service use in 23,317 Canadian Longitudinal Study on Aging Tracking and Comprehensive Follow-up 1 survey respondents age 65+ from 2015-2018 by loneliness (lonely, not lonely) and gender (men, women, gender diverse)

| **Variables** | **Total** | **Lonely (n=23,317)** | | **Std. difference** | **Men (n=11,613)** | | **Women (n=11,675)** | | **Gender Diverse (n=17)** | |
| --- | --- | --- | --- | --- | --- | --- | --- | --- | --- | --- |
|  |  |  |  |  | **Lonely** | | **Lonely** | | **Lonely** | |
|  |  | **No** | **Yes** |  | **No** | **Yes** | **No** | **Yes** | **No** | **Yes** |
|  | **N (%)** | **N (%)** | **N (%)** |  | **N (%)** | **N (%)** | **N (%)** | **N (%)** | **N (%)** | **N (%)** |
| **Total** | 23317 | 18002 | 5315 |  | 9317 | 2296 | 8661 | 3014 | 13 | 4 |
| **ED visit** |  |  |  |  |  |  |  |  |  |  |
| Yes | 5755(24.7) | 4191(23.3) | 1564(29.4) | -0.14 | 2167(23.3) | 672(29.3) | 2019(23.3) | 890(29.5) | 3(23.1) | 2(50) |
| No | 17484(75) | 13760(76.4) | 3724(70.1) | 0.14 | 7129(76.5) | 1609(70.1) | 6613(76.4) | 2112(70.1) | 10(76.9) | 2(50) |
| **Education** |  |  |  |  |  |  |  |  |  |  |
| Less than university | 11913(51.1) | 9095(50.5) | 2818(53) | -0.05 | 4183(44.9) | 1084(47.2) | 4901(56.6) | 1733(57.5) | 5(38.5) | 1(25) |
| University or higher | 9375(40.2) | 7495(41.6) | 1880(35.4) | 0.13 | 4465(47.9) | 988(43) | 3019(34.9) | 891(29.6) | 7(53.8) | 0(0) |
| **Ethnicity** |  |  |  |  |  |  |  |  |  |  |
| All else | 685(2.9) | 484(2.7) | 201(3.8) | -0.06 | 303(3.3) | 106(4.6) | 181(2.1) | 95(3.2) | 0(0) | 0(0) |
| White | 22632(97.1) | 17518(97.3) | 5114(96.2) | 0.06 | 9014(96.7) | 2190(95.4) | 8480(97.9) | 2919(96.8) | 13(100) | 4(100) |
| **Geographic region** |  |  |  |  |  |  |  |  |  |  |
| Rural | 2331(10) | 1815(10.1) | 516(9.7) | 0.01 | 982(10.5) | 249(10.8) | 827(9.5) | 267(8.9) | 3(23.1) | 0(0) |
| Urban | 20986(90) | 16187(89.9) | 4799(90.3) | -0.01 | 8335(89.5) | 2047(89.2) | 7834(90.5) | 2747(91.1) | 10(76.9) | 4(100) |
| **Income** |  |  |  |  |  |  |  |  |  |  |
| <$20,000 | 1309(5.6) | 777(4.3) | 532(10) | -0.22 | 210(2.3) | 159(6.9) | 565(6.5) | 370(12.3) | 1(7.7) | 3(75) |
| $20,000-<$50,000 | 7097(30.4) | 5045(28) | 2052(38.6) | -0.23 | 2126(22.8) | 778(33.9) | 2911(33.6) | 1274(42.3) | 7(53.8) | 0(0) |
| $50,000+ | 12885(55.3) | 10716(59.5) | 2169(40.8) | 0.38 | 6466(69.4) | 1184(51.6) | 4240(49) | 983(32.6) | 5(38.5) | 1(25) |
| **Marital status** |  |  |  |  |  |  |  |  |  |  |
| Single, never married | 1542(6.6) | 1028(5.7) | 514(9.7) | -0.15 | 419(4.5) | 238(10.4) | 602(7) | 276(9.2) | 2(15.4) | 0(0) |
| Divorced/separated | 4326(18.6) | 2793(15.5) | 1533(28.8) | -0.33 | 633(6.8) | 472(20.6) | 2157(24.9) | 1058(35.1) | 3(23.1) | 3(75) |
| Married/common law | 14556(62.4) | 12307(68.4) | 2249(42.3) | 0.54 | 7687(82.5) | 1224(53.3) | 4608(53.2) | 1023(33.9) | 7(53.8) | 1(25) |
| Widowed | 2879(12.3) | 1862(10.3) | 1017(19.1) | -0.25 | 572(6.1) | 362(15.8) | 1288(14.9) | 655(21.7) | 1(7.7) | 0(0) |
| **Living alone** |  |  |  |  |  |  |  |  |  |  |
| No | 15672(67.2) | 13071(72.6) | 2601(48.9) | 0.50 | 7800(83.7) | 1308(57) | 5258(60.7) | 1291(42.8) | 8(61.5) | 1(25) |
| Yes | 7645(32.8) | 4931(27.4) | 2714(51.1) | -0.50 | 1517(16.3) | 988(43) | 3403(39.3) | 1723(57.2) | 5(38.5) | 3(75) |
| **Number of chronic conditions** |  |  |  |  |  |  |  |  |  |  |
| <4 | 17281(74.1) | 13900(77.2) | 3381(63.6) | 0.30 | 7867(84.4) | 1689(73.6) | 6014(69.4) | 1691(56.1) | 12(92.3) | 1(25) |
| 4+ | 6036(25.9) | 4102(22.8) | 1934(36.4) | -0.30 | 1450(15.6) | 607(26.4) | 2647(30.6) | 1323(43.9) | 1(7.7) | 3(75) |
| **Functional impairment** |  |  |  |  |  |  |  |  |  |  |
| None | 18046(77.4) | 14477(80.4) | 3569(67.1) | 0.31 | 8214(88.2) | 1784(77.7) | 6247(72.1) | 1783(59.2) | 10(76.9) | 1(25) |
| Mild/moderate/severe/total | 4585(19.7) | 3005(16.7) | 1580(29.7) | -0.31 | 1001(10.7) | 481(20.9) | 1998(23.1) | 1096(36.4) | 2(15.4) | 3(75) |
| **Self-rated mental health** |  |  |  |  |  |  |  |  |  |  |
| Poor | 141(0.6) | 50(0.3) | 91(1.7) | -0.14 | 24(0.3) | 43(1.9) | 26(0.3) | 48(1.6) | 0(0) | 0(0) |
| Fair/Good/Very Good/Excellent | 23130(99.2) | 17925(99.6) | 5205(97.9) | 0.15 | 9281(99.6) | 2247(97.9) | 8621(99.5) | 2954(98) | 13(100) | 3(75) |
| **Number of depressive symptoms** |  |  |  |  |  |  |  |  |  |  |
| Median (Q1-Q3) | 19(17-20) | 17(15-20) | 19(17-21) |  | 19(18-21) | 18(16-20) | 19(17-20) | 17(14-19) | 19(17-21) | 12(10-18) |
| <10 | 371(1.6) | 170(0.9) | 201(3.8) | -0.19 | 67(0.7) | 64(2.8) | 103(1.2) | 136(4.5) | 0(0) | 1(25) |
| 10+ | 22340(95.8) | 17381(96.6) | 4959(93.3) | 0.15 | 9031(96.9) | 2157(93.9) | 8328(96.2) | 2798(92.8) | 12(92.3) | 3(75) |
| **Number of social contacts** |  |  |  |  |  |  |  |  |  |  |
| Median (Q1-Q3) | 2(2-3) | 3(2-3) | 2(2-3) |  | 4-3-5) | 4(3-5) | 4(3-4) | 3(2-4) | 3(2-3) | 3(2-4) |
| High contact (4-5) | 11279(48.4) | 9054(50.3) | 2225(41.9) | 0.17 | 4479(48.1) | 872(38) | 4568(52.7) | 1351(44.8) | 3(23.1) | 1(25) |
| Moderate contact (2-3) | 10060(43.1) | 7585(42.1) | 2475(46.6) | -0.09 | 4046(43.4) | 1110(48.3) | 3524(40.7) | 1363(45.2) | 10(76.9) | 2(50) |
| Low contact (0-1) | 1977(8.5) | 1362(7.6) | 615(11.6) | -0.14 | 792(8.5) | 314(13.7) | 568(6.6) | 300(10) | 0(0) | 1(25) |
| **Number of social activities** |  |  |  |  |  |  |  |  |  |  |
| Median (Q1-Q3) | 2(2-2) | 2(2-3) | 2(1-2) |  | 3(3-2) | 2(1-3) | 3(2-4) | 3(2-3) | 2(2-3) | 1(1-2) |
| High participation (4-5) | 5605(24) | 4583(25.5) | 1022(19.2) | 0.15 | 2145(23) | 383(16.7) | 2433(28.1) | 639(21.2) | 3(23.1) | 0(0) |
| Moderate participation (2-3) | 13158(56.4) | 10315(57.3) | 2843(53.5) | 0.08 | 5403(58) | 1232(53.7) | 4899(56.6) | 1609(53.4) | 7(53.8) | 1(25) |
| Low participation (0-1) | 4186(18) | 2834(15.7) | 1352(25.4) | -0.24 | 1653(17.7) | 628(27.4) | 1176(13.6) | 721(23.9) | 3(23.1) | 3(75) |
| **Anxiety** |  |  |  |  |  |  |  |  |  |  |
| No | 21044(90.3) | 16518(91.8) | 4526(85.2) | 0.21 | 8710(93.5) | 1993(86.8) | 7786(89.9) | 2529(83.9) | 12(92.3) | 3(75) |
| Yes | 1673(7.2) | 1036(5.8) | 637(12) | -0.22 | 383(4.1) | 229(10) | 653(7.5) | 407(13.5) | 0(0) | 1(25) |
| **Family doctor in last 12 months** |  |  |  |  |  |  |  |  |  |  |
| No | 1278(5.5) | 979(5.4) | 299(5.6) | -0.01 | 518(5.6) | 133(5.8) | 459(5.3) | 166(5.5) | 1(7.7) | 0(0) |
| Yes | 21985(94.3) | 16986(94.4) | 4999(94.1) | 0.01 | 8784(94.3) | 2151(93.7) | 8181(94.5) | 2843(94.3) | 12(92.3) | 4(100) |

Supplementary Table 4. Logistic regression model results to test associations between loneliness and ED visit in previous 12 months in 21,201 Canadian Longitudinal Study on Aging Tracking and Comprehensive Follow-up 1 (2015-2018) survey respondents aged less than 65 years

| **Variables** | **Odds Ratio (95% CI)**  **N=21,201** | |
| --- | --- | --- |
|  | **Unadjusted** | **Adjusted (weighted)*** |
| **Lonely** (ref: not lonely) | 1.45(1.34-1.56) | 1.11(1.01-1.22) |

*Adjusted for education, ethnicity, geographic region, gender, income, marital status, living alone, chronic conditions, functional impairment, self-rated mental health, depressive symptoms, anxiety, social contacts, social activities, family physician visit.

Supplementary Table 5. Logistic regression model results to test associations between loneliness and ED visit in previous 12 months in 23,317 Canadian Longitudinal Study on Aging Tracking and Comprehensive Follow-up 1 (2015-2018) survey respondents aged 65+

| **Variables** | **Odds Ratio (95% CI)**  **N=23,317** | |
| --- | --- | --- |
|  | **Unadjusted** | **Adjusted (weighted)*** |
| **Lonely** (ref: not lonely) | 1.38(1.29-1.48) | 1.14(1.04-1.25) |

*Adjusted for education, ethnicity, geographic region, gender, income, marital status, living alone, chronic conditions, functional impairment, self-rated mental health, depressive symptoms, anxiety, social contacts, social activities, family physician visit.
